# Overexpression of connexin 43 using a retroviral vector improves electrical coupling of skeletal myoblasts with cardiac myocytes *in vitro*

**DOI:** 10.1186/1471-2261-6-25

**Published:** 2006-06-06

**Authors:** Oleg Tolmachov, Yu-Ling Ma, Michael Themis, Pravina Patel, Hilmar Spohr, Kenneth T MacLeod, Nina D Ullrich, Yvonne Kienast, Charles Coutelle, Nicholas S Peters

**Affiliations:** 1 Section of Molecular and Cellular Medicine, Division of Biomedical Sciences, Faculty of Life Sciences, Imperial College London, London, UK; 2 Department of Cardiac Electrophysiology, National Heart and Lung Institute, Imperial College London at St. Mary's Hospital, London, UK; 3 Department of Cardiac Medicine, National Heart and Lung Institute, Imperial College London, London, UK

## Abstract

**Background:**

Organ transplantation is presently often the only available option to repair a damaged heart. As heart donors are scarce, engineering of cardiac grafts from autologous skeletal myoblasts is a promising novel therapeutic strategy. The functionality of skeletal muscle cells in the heart milieu is, however, limited because of their inability to integrate electrically and mechanically into the myocardium. Therefore, in pursuit of improved cardiac integration of skeletal muscle grafts we sought to modify primary skeletal myoblasts by overexpression of the main gap-junctional protein connexin 43 and to study electrical coupling of connexin 43 overexpressing myoblasts to cardiac myocytes *in vitro*.

**Methods:**

To create an efficient means for overexpression of connexin 43 in skeletal myoblasts we constructed a bicistronic retroviral vector MLV-CX43-EGFP expressing the human connexin 43 cDNA and the marker EGFP gene. This vector was employed to transduce primary rat skeletal myoblasts in optimised conditions involving a concomitant use of the retrovirus immobilising protein RetroNectin^® ^and the polycation transduction enhancer Transfectam^®^. The EGFP-positive transduced cells were then enriched by flow cytometry.

**Results:**

More than four-fold overexpression of connexin 43 in the transduced skeletal myoblasts, compared with non-transduced cells, was shown by Western blotting. Functionality of the overexpressed connexin 43 was demonstrated by microinjection of a fluorescent dye showing enhanced gap-junctional intercellular transfer in connexin 43 transduced myoblasts compared with transfer in non-transduced myoblasts. Rat cardiac myocytes were cultured in multielectrode array culture dishes together with connexin 43/EGFP transduced skeletal myoblasts, control non-transduced skeletal myoblasts or alone. Extracellular field action potential activation rates in the co-cultures of connexin 43 transduced skeletal myoblasts with cardiac myocytes were significantly higher than in the co-cultures of non-transduced skeletal myoblasts with cardiac myocytes and similar to the rates in pure cultures of cardiac myocytes.

**Conclusion:**

The observed elevated field action potential activation rate in the co-cultures of cardiac myocytes with connexin 43 transduced skeletal myoblasts indicates enhanced cell-to-cell electrical coupling due to overexpression of connexin 43 in skeletal myoblasts. This study suggests that retroviral connexin 43 transduction can be employed to augment engineering of the electrocompetent cardiac grafts from patients' own skeletal myoblasts.

## Background

There is only minor potential for cell renewal in the adult myocardium [1]. Loss of myocardial cells due to cardiac disease results in impairment of cardiac contractile and electrical function, and despite currently available medical therapies the mortality rate remains substantial and worsens with deteriorating myocardial function [2]. Cardiac transplantation is currently the treatment offered to selected patients with end-stage left ventricular dysfunction, but due to limited donor supply and resources, this mode of therapy will remain confined to a minority of patients [3].

Cell transplantation is a promising means of repairing damaged myocardium. A number of different cell types and their combinations are under investigation for transplantation to the ventricular myocardium, including neonatal or fetal cardiomyocytes, autologous skeletal myoblasts, fibroblasts, hematopoetic stem cells, and embryonic stem cell-derived cells [4-6]. Transplanted cells have been widely reported to engraft into the host myocardium, but with variability in the degree of differentiation and integration with the host tissue [7-9].

Owing to immunological, ethical and practical advantages over some of the other cell types, transplantation of autologous skeletal myoblasts for myocardial repair was the first to undergo clinical trials [10,11]. Although modest improvements of cardiac function have been reported, ventricular tachycardia was observed in a number of patients indicating absence of electrical incorporation of the grafted cells into the host myocardium. In general, the results obtained from cell, animal and human studies have indicated that the implanted myoblasts showed fusion and differentiated into multinucleated myotubes, did not transdifferentiate to cardiac myocytes and did not couple with the host cardiac myocytes. It is possible that lack of electrical coupling of the implanted cells with the host myocytes is the key factor blocking adequate functional incorporation of the grafted skeletal myoblasts into the beating cardiac muscle [12]. Indeed, the major myocardial gap junctional protein connexin 43 is not expressed in mature skeletal myotubes [13]. We therefore addressed the hypothesis that connexin 43 overexpression by retroviral transduction of the skeletal myoblasts can enhance their electrical integration with cardiac myocytes in co-culture.

## Methods

### *E. coli *strain, transformation and bacterial culture conditions

*E. coli *strain DH10B F^- ^*mcrA *Δ*(mrr-hsdRMS-mcrBC) *ϕ*80lacZ*Δ*M15 *Δ*lacX74 recA1 endA1 araD139 *Δ*(ara, leu)7697 galU galK *λ^- ^*rpsL(Str^R^) nupG *(Invitrogen) was used as a plasmid host. This strain was transformed by electroporation using the Gene Pulser^® ^II apparatus (BioRad). Bacterial clones were cultured in the LB medium supplemented with appropriate antibiotics [14].

### Plasmids, oligonucleotides, PCR and sequencing

Plasmid pGF1 containing human connexin 43 cDNA [15] and primers CX-NHE 5'-TACTGCAGAA GCTAGCAGCC GCCACCATGG GTGACTGGAG CGCCTTAGGC-3' and CX-BAM 5'-TGTTACTATA GGATCCCTAG ATCTCCAGGT CATCAGGCC-3' were employed to amplify the connexin 43 gene by PCR using high fidelity Pfx DNA polymerase (Invitrogen). The forward PCR primer CX-NHE included a recognition sequence for *Nhe*I followed by the consensus extended Kozak sequence 5'-GCCGCCACC-3'. The reverse PCR primer CX-BAM contained a *Bam*HI recognition sequence at its 5' end. The obtained PCR product was cut by *Nhe*I and *Bam*HI and inserted into the *Nhe*I and *Bam*HI digested plasmid pIRES2EGFP (Clontech) to produce the new plasmid pCX43-IRES-EGFP. Then the entire connexin 43 cDNA insert was sequenced (Advanced Biotechnology Centre, Imperial College London) using primers CX-SEQ2 5'-TTGCTGCGAACCTACATCATCAGT-3', CX-SEQ3 5'-TTTCAATGGC TGCTCCTCACCAAC-3' and CX-SEQ4 5'-GAAGATGGTTTTCTCCGTGGGGCGAGA-3' to confirm its faithful reproduction by PCR.

**Figure 1:**
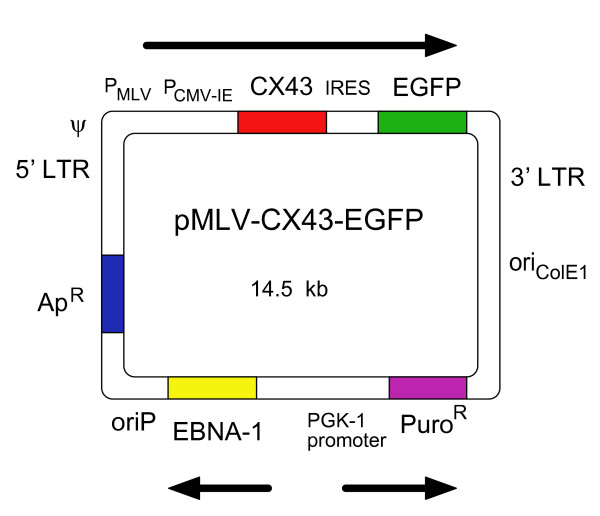
**Plasmid pMLV-CX43-EGFP containing the DNA sequence for the retroviral vector MLV-CX43-EGFP**. A retroviral backbone plasmid, pMLV-CX43-EGFP, co-expressing human connexin 43 cDNA and the EGFP gene, was generated on the basis of the retroviral backbone plasmid pLZRS-LacZ(A). We used the internal ribosome entry site (IRES) to arrange the connexin 43 sequence and the downstream EGFP marker gene within one single transcription unit under control of a tandem of MLV LTR promoter and CMV immediate early promoter. The obtained plasmid pMLV-CX43-EGFP contains the Epstein-Barr virus EBNA-1-oriP segment driving episomal replication in the producer cell line and, thus, improving chances for selection of a high titre virus producing clone. The EGFP marker of the retroviral vector simplifies identification of transduced cells and is useful for evaluation of the viral vector titre.

The CX43-IRES-EGFP bicistronic expression cassette was excised using *Ase*I and *Not*I to include the CMV promoter but to exclude the SV40 polyadenylation signal. This fragment was blunt-ended by the Klenow fragment of *Escherichia coli *DNA polymerase I (KF) and inserted between KF-polished *Eco*RI and *Not*I ends of the digested retroviral MLV vector plasmid pLZRS-LacZ(A) [16]. The resultant retroviral vector plasmid was called pMLV-CX43-EGFP (Figure 1).

### Tissue culture techniques and construction of the producer cell line generating retroviral vector MLV-CX43-EGFP

Packaging cells, virus producer cells, and immortalised L6 rat myoblasts (ATCC CRL-1458) were grown in DMEM with glutamax-I supplemented with 10% FCS (Invitrogen) at 37°C with 5% CO_2 _in air.

To generate retroviral producer cell lines, the amphotropic MLV packaging cell line TEFLYA [17] was transfected with the obtained plasmid pMLV-CX43-EGFP. Transfection was performed using Lipofectamin^® ^(Invitrogen) as recommended by the supplier. Transfected cells were selected in the medium supplemented with puromycin (10 μg/ml). Different dilutions of the trypsinised transfected cells were prepared and seeded onto 96 well plates. 267 individual clones were produced and expanded in 24 well plates. The clones were screened using an inverted fluorescence microscope (Leitz) for EGFP expression and also for viral vector production by transduction of L6 rat skeletal myoblasts. 39 clones (14.6%) were fluorescent of which 17 clones (6.4%) produced virus. Screening of supernatants from 267 potential producer cell lines revealed a broad variation in the titre of produced virus, determined by transduction of L6 rat skeletal myoblasts in the presence of 10 μg/ml of Polybrene™. Clones A113, A189 and A247 produced virus with the highest end-point titre (1 × 10^6 ^TU/ml in the freshly collected virus-containing medium). Clone A247 was used as a source of the MLV-CX43-EGFP virus vector for further experiments. Virus vector preparations were produced by centrifugation of culture supernatants from producer cells at 5000 rpm for 10 min and filtration of the obtained fluid through 0.8 μm filters (Sartorius). The virus vector preparations were stored frozen at -80°C.

### Isolation and culture of skeletal myoblasts and cardiac myocytes

The manipulations of animals in this work conform to UK Home Office guidelines. Primary rat skeletal myoblasts were isolated from the hind leg muscles of adult male Wistar rats. The muscle slices were digested in 0.25% pancreatin, 1% trypsin for 1 hour with occasional agitation. The isolated cells were collected by filtering through 70 μm nylon cell strainers (Falcon). Counter-selection against fibroblasts was accomplished by 2 rounds of differential adhesion on collagen coated tissue culture flasks (40 min at 37°C for each adhesion step). Primary myoblasts were cultured in a CO_2 _incubator at 37°C in DMEM with glutamax-I (Invitrogen) supplemented with 20% FCS and further purified by sorting using paramagnetic beads (Dynal Biotech) coated with antibody H36 against myoblast specific α-7 integrin [18].

Cardiac myocytes were isolated from neonatal rats. Freshly excised ventricles were dissociated in trypsin-EDTA (Invitrogen) and the dispersed cells were suspended in the culture medium, a 4:1 mixture of DMEM and M199 media (Invitrogen) supplemented with 15% horse serum and 5% FCS. The cell suspension was pre-plated to separate fibroblasts from myocytes as described for skeletal myoblasts above. The myocytes remaining in the suspension were cultured in a CO_2 _incubator at 37°C.

### Retroviral transduction of primary skeletal myoblasts and FACS analysis

**Figure 2:**
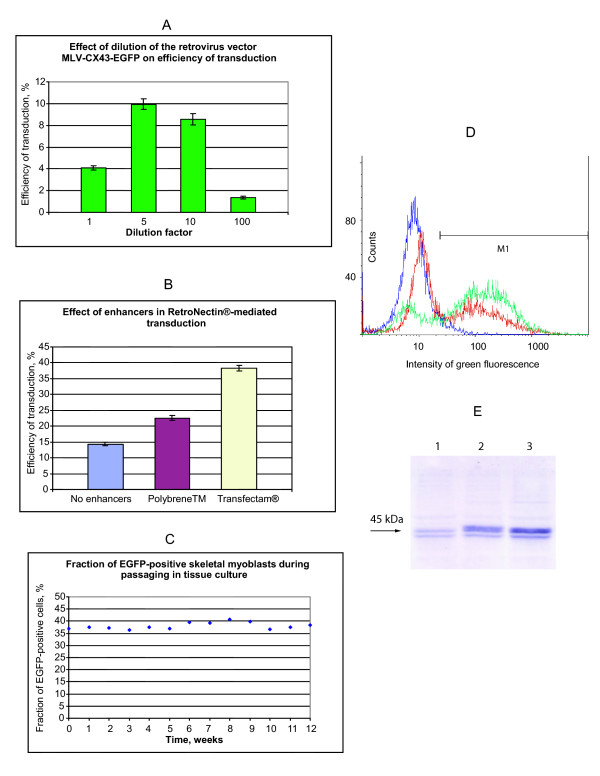
**Transduction of primary rat skeletal myoblasts by the retroviral vector MLV-CX43-EGFP and resultant overexpression of connexin 43**. Connexin 43 and EGFP were expressed from a single bicistronic transcription unit. **(A) **Transduction of primary rat skeletal myoblasts using various dilutions of the MLV-CX43-EGFP viral vector preparation. Medium in the wells of a 24-well plate containing non-confluent primary myoblasts was aspirated and the wells were filled with 1 ml of the non-diluted and diluted viral vector suspensions supplemented with Polybrene™ (10 μg/ml). After 48 hours the cells were trypsinised, washed in complete DMEM medium, resuspended in PBS and used for FACS analysis to determine the efficiency of transfection relying on EGFP expression. The obtained data were averaged among 3 wells for each virus vector dilution. **(B) **Concomitant use of RetroNectin^®^-mediated viral vector concentration and polycation transduction enhancement for delivery of the connexin 43 and EGFP genes into primary skeletal myoblasts using MLV-CX43-EGFP vector. Transduction experiments were performed in 6-well plates containing non-confluent myoblasts in the presence of the polycation Polybrene™ (10 μg/ml), in the presence of the polycation Transfectam^® ^(5 μg/ml) or without addition of polycations. The efficiency of transduction was determined using the EGFP marker from the FACS subtraction plots using non-transduced primary skeletal myoblasts as a control, non-fluorescent cell population. The data were averaged among 6 individual transduction experiments. **(C) **Fractions of EGFP-positive cells in the population of connexin 43/EGFP transduced primary skeletal myoblasts during continuous passaging. The cells were split and re-seeded in the ratio 1:10 each week with concomitant FACS measurement of the fraction of the EGFP-expressing cells. **(D) **Results of FACS analysis of populations of primary skeletal myoblasts plotted as histograms (x-axis – intensity of green fluorescence, y-axis – counts of cells). Purple graph corresponds to non-transduced primary skeletal myoblasts, red graph corresponds to the primary skeletal myoblasts after RetroNectin^®^-mediated Transfectam^®^-enhanced transduction by MLV-CX43-EGFP, green graph represents primary skeletal myoblasts, which were preparatively sorted for EGFP expression after transduction by MLV-CX43-EGFP. **(E) **Western blotting analysis of connexin 43 expression in connexin 43 transduced skeletal myoblasts (after EGFP-based preparative sorting). The cell extracts were analysed by electrophoresis in a SDS-PAGE gel and immunoblotting using anti-connexin 43 antibody. Lane 1 corresponds to non-transduced primary skeletal myoblasts, lane 2 corresponds to connexin 43 transduced primary skeletal myoblasts, lane 3 corresponds to connexin 43 transduced primary skeletal myoblasts, which were passaged 13 weeks in tissue culture.

Filtered preparation of the MLV-CX43-EGFP was poured into RetroNectin^® ^(TaKaRa) coated plates for the virions to attach. Myoblasts were loaded onto the immobilised viral vector particles in the DMEM supplemented with 20% FCS and, optionally, 5 μg/ml Transfectam^® ^(dioctadecylamidoglycyl spermine, DOGS, Promega) or 10 μg/ml Polybrene™ (hexadimethrine bromide, Sigma). The proportion of the transduced cells was determined by FACS analysis on a FACSCalibur machine (Becton Dickinson) relying on expression of the EGFP transgene. The total number of counted cells was 10000 in all FACS measurements. The transduced cells were sorted using a preparative FACS machine (Becton Dickinson FACS DIVA cell sorter) to produce a cell population with more than 70% of the cells expressing the EGFP marker (Figure 2D). The sorted cells were passaged once and their myogenic nature was confirmed by immunoconfocal analysis with a monoclonal anti-desmin antibody (clone DE-U-10, product number D033 from Sigma-Aldrich) as shown in Additional file 2: Additional_file_2.pdf.

The obtained population of connexin 43 transduced myoblasts was frozen in liquid nitrogen in the DMEM containing 20% FCS and 10% DMSO. Aliquots of the frozen transduced skeletal myoblasts were used to seed subcultures for Western blotting analysis, dye transfer and electrophysiological experiments.

### Western blotting analysis of connexin 43 expression

Quantitative immunoblotting was performed to confirm overexpression of connexin 43 in the skeletal myoblasts containing the connexin 43 transgene. Seeded cells were allowed to expand until they reached complete confluence (7 days). Then the cells were scraped off, homogenised in the lysis buffer SB20 (20% SDS and 0.15 M Tris-HCl pH 6.8) and sonicated to shear the genomic DNA. The protein concentration was determined and adjusted to 0.5 mg/ml. Samples with 5.0 μg of total protein were resolved by SDS-PAGE on a 12.5% gel and transferred onto a polyvinylidene difluoride membrane. The mouse primary antibody against connexin 43 (Chemicon), the secondary alkaline phosphatase-conjugated anti-mouse antibody (Pierce) and the proprietary alkaline phosphatase substrate (Promega) were used to detect connexin 43. The protein bands were quantified by densitometry. Two independent cell cultivation experiments were performed and two independent gels were analysed by Western blotting for each cultivation experiment.

### Microinjection and intercellular transfer of a fluorescent dye

Skeletal myoblasts transduced with the MLV-CX43-EGFP vector, control non-transduced skeletal myoblasts and co-cultures of these cells with cardiac myocytes were grown in culture dishes to ~80% confluence. The culture dishes were then placed onto the stage of an inverted fluorescence microscope. Microelectrodes with resistance 50–60 MΩ were loaded with a 5% solution of Cascade Blue derivative 8-methoxypyrene-trisulphonic acid (Molecular Probes, Oregon, USA) and were back-filled with 1 M LiCl. The dye was iontophoresed into each cell by 4–6 nA current for 2 min following impalement of a cell by the electrode. Dye transfer to the adjacent cells was recorded using a Nikon digital camera (Coolpix 990). Dye transfer images were captured 2 min after injection. The images were used to score the number of neighbouring cells to which the dye was transferred from each injected cell.

### Electrophysiological measurements in cell co-cultures

Cell culture dishes incorporating a group of 60 embedded unipolar electrodes with diameter 30 μm with interelectrode distances of 100 μm (Multielectrode Array, MCS GmbH, Reutlingen, Germany) were used to study and compare the electrical integration of skeletal myoblasts (connexin 43/EGFP transduced and non-transduced) in co-cultures with cardiac myocytes. Five groups of cell cultures were under investigation: 1) cardiac myocytes alone; 2) skeletal myoblasts alone; 3) connexin 43 transduced skeletal myoblasts alone; 4) cardiac myocytes co-cultured in a ratio of 4:1 with connexin 43 transduced skeletal myoblasts; 5) cardiac myocytes co-cultured in the same ratio with non-transduced skeletal myoblasts. To establish co-cultures, cardiac myocytes were seeded in the multielectrode array dishes (1 million cells per dish) to allow the cells to settle. The cells were cultured in a medium composed of a 4:1 mixture of DMEM and M199 media (Invitrogen) supplemented with 15% horse serum and 5% FCS. At day 2 after the initial seeding, connexin 43 transduced skeletal myoblasts or non-transduced skeletal myoblasts (0.25 million cells per dish) were added to cardiac myocytes. At day 3, the cells reached confluence with both skeletal and cardiac cells distinguishable under the microscope. Then MEA dishes were placed onto the recording system and an extracellular stimulatory current was applied in 10 evenly spaced pulses (80 μA, 5 ms) during 10 s time interval. The stimulatory pulses were delivered to the cells by a pair of electrodes located outside the 60-electrode array. To register FAP, electrograms (potential against time) were recorded for 10 s. The FAP activation rate (that is, frequency of FAP firing) was determined from the discrete spikes on the electrograms with amplitude > 500 μV and duration > 5 ms using the spike sorter of the MC-Rack data analyser (Microcal Software, Northampton, MA, USA). The FAP activation rate data obtained from the 60 electrodes were then averaged.

### Statistical analysis

The data are presented as the mean value and its standard error (mean ± standard error, M ± SE). The following formula was used to compute average standard error (ASE) of the ratio of two means (M_1 _± SE_1_, M_2 _± SE_2_): ASE = (M_1_/M_2_)*(SE_1_^2^/M_1_^2^+SE_2_^2^/M_2_^2^)^1/2^. The significance of the differences between experimental groups was estimated using unpaired t-test.

## Results

### Construction of the retroviral connexin 43 vector MLV-CX43-EGFP and optimisation of myoblast transduction conditions

To be able to deliver the connexin 43 gene to skeletal myoblasts we constructed an amphotropic retroviral vector MLV-CX43-EGFP, co-expressing human connexin 43 cDNA and the EGFP marker gene (Figure 1).

The passaging time of primary skeletal myoblasts in tissue culture before transplantation is limited because of clinical considerations. Therefore, it is important to achieve maximum cell transduction efficiency with a minimal number of exposures of the myoblasts to the viral vector. Amphotropic MLV vector preparations commonly contain substances, which severely limit the efficiency of transduction. In an attempt to minimise the adverse effect of the transduction inhibitors, we used various dilutions of the MLV-CX43-EGFP viral vector preparation with end-point titre 2 × 10^5 ^TU/ml for infection of the primary rat skeletal myoblasts in the presence of 5 μg/ml of the commonly used transduction enhancer polycation Polybrene™ (hexadimethrine bromide). The highest efficiency of transduction (9.94 ± 0.50%) was achieved with 5-fold dilution of the viral vector and not with undiluted vector, confirming contamination of the vector preparation by transduction inhibitors (Figure 2A). To increase efficiency of transduction by the MLV-CX43-EGFP vector, we tested RetroNectin^®^-mediated virion capture [19] to immobilise the vector particles on a plastic surface and, thus, to get rid of the RetroNectin^®^-unbound portion of the transduction inhibitors. We compared the standard RetroNectin^®^-mediated transduction protocol, which is performed without polycation transduction enhancers, to RetroNectin^®^-mediated transduction in the presence of Polybrene™ or, alternatively, in the presence of lipopolyamine Transfectam^® ^(dioctadecylamidoglycyl spermine, [20]). The obtained transduction efficiency data are summarised in Figure 2B. Employment of Transfectam^® ^to enhance transduction of primary skeletal myoblasts by RetroNectin^®^-immobilized MLV-CX43-EGFP vector allowed transduction of 38.30 ± 0.89% cells. In contrast, the efficiency of transduction was 22.56 ± 0.75% in the RetroNectin^®^/Polybrene™ experiments and 14.34 ± 0.60% when RetroNectin^®^-immobilised virus was used without addition of a polycation substance.

To estimate the stability of connexin 43 expression, one part of the transduced population of skeletal myoblasts was stored frozen in liquid nitrogen and another part was used for continuous passaging. During 12-week cultivation, the cells were split and re-seeded in the ratio 1:10 each week with simultaneous FACS analysis of the EGFP transgene expression. The percentage of EGFP-positive cells stayed practically constant during passaging *in vitro *indicating absence of the EGFP transgene shut-down and suggesting the absence of shut-down of the linked connexin 43 transgene (Figure 2C).

The passaged and the stored populations of transduced myoblasts were then used for preparative FACS to enrich EGFP expressing cells to more than 70% (Figure 2D).

Western blotting analysis showed a 4.85 ± 0.25 times overexpression of connexin 43 in the population of sorted non-passaged MLV-CX43-EGFP transduced skeletal myoblasts compared with control non-transduced skeletal myoblasts. Confirming longevity of connexin 43 expression in tissue culture, Western blotting analysis showed a 4.71 ± 0.23 times overexpression of connexin 43 in the population of passaged sorted skeletal myoblasts compared with non-transduced skeletal myoblasts (Figure 2E).

### Connexin 43 overexpression enhances intercellular dye transfer between skeletal myoblasts

**Figure 3:**
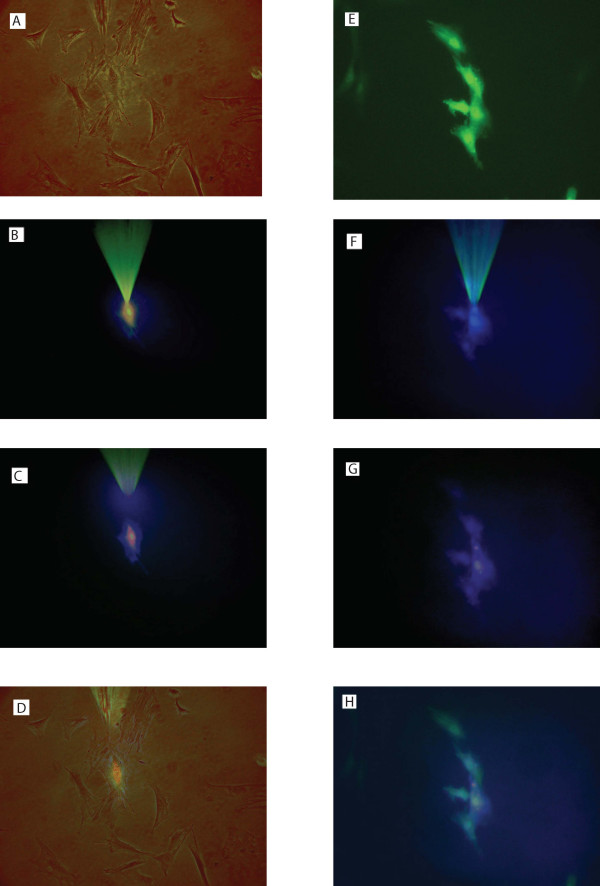
**Intercellular fluorescent dye microinjection in connexin 43 and EGFP transduced and non-transduced primary skeletal myoblasts**. Cascade Blue derivative 8-methoxypyrene-trisulphonic acid was used as a fluorescent dye. Panel 'ABCD' shows fluorescent dye microinjection experiment in a culture of non-transduced primary skeletal myoblasts. Panel 'EFGH' shows fluorescent dye microinjection experiment in a culture of connexin 43 and EGFP transduced primary skeletal myoblasts. All pictures were taken using 400 times instrumental magnification. Blue fluorescence was visualised using Nikon filter block UV-1A (DM400). Green fluorescence was visualised using a standard 'FITC' filter block. **(A) **A phase contrast image of a group of adjacent non-transduced skeletal myoblasts. **(B) **An image of the injection microelectrode inserted into one of the adjacent cells under UV light. **(C) **An image of the same group of the cells under UV light after 2 min, no dye transfer. **(D) **An overlay of 'A' and 'C'. **(E) **An image of a group of adjacent connexin 43 and EGFP transduced skeletal myoblasts under UV light before dye injection (green fluorescence confirms EGFP expression). **(F) **An image of the injection microelectrode inserted into one of the adjacent cells under UV light. **(G) **An image of the same group of cells under UV light after 2 min, the dye is transferred to adjacent cells. **(H) **An overlay of 'E' and 'G'.

To show that connexin 43 overexpression has improved intercellular communication in skeletal myoblasts, we injected fluorescent dye 8-methoxypyrene-trisulphonic acid (a Cascade Blue derivative) into individual cells in pure cultures of connexin 43 transduced and non-transduced skeletal myoblasts (Figure 3). 13 injections of the dye were carried out in the cultures of connexin 43 transduced skeletal myoblasts, 10 of them (77%) resulted in dye migration to the neighbouring cells. In contrast, just 3 out of 11 (27%) injections led to intercellular dye spread in non-transduced skeletal myoblasts. The number of cells to which the dye permeated from the injected cell varied and was 1.38 ± 0.33 for connexin 43 transduced cells and 0.27 ± 0.14 for non-transduced ones. Thus, enhanced dye spread to neighbouring cells was observed in connexin 43 transduced skeletal myoblasts compared with non-transduced ones (P < 0.05), indicating a higher density of gap junctions in the cells overexpressing connexin 43.

### Connexin 43 overexpression in skeletal myoblasts improves electrical coupling in co-cultures of cardiac myocytes and skeletal myoblasts

**Figure 4:**
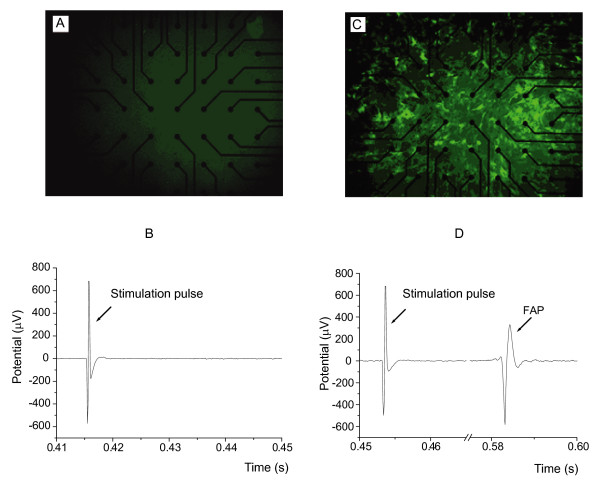
**Multi-electrode array recording in co-cultures of primary skeletal myoblasts with cardiac myocytes**. Microscopic images of the multi-electrode array (MEA) dishes containing co-cultures of cardiac myocytes with non-transduced **(A) **or connexin 43/EGFP transduced **(C) **primary skeletal myoblasts. The images were taken under UV light with 100 times magnification. Skeletal myoblasts transduced with MLV-CX43-EGFP vector expressed the EGFP marker and, therefore, were fluorescent. Individual recordings (x-axis – time in s, y-axis – potential in μV) from the electrode No. 33 of the MEA in co-culture of cardiac myocytes with non-transduced skeletal myoblasts **(B) **and in co-culture of cardiac myocytes with connexin 43/EGFP transduced skeletal myoblasts **(D)**. These electrograms show the last stimulatory pulse in the series of 10 and the first resultant FAP **(D) **or absence of it **(B)**. Ten stimulatory current pulses were applied with the frequency of 1 Hz.

Co-cultures of skeletal myoblasts and cardiac myocytes were established to mimic *in vivo *transplantation of skeletal myoblasts to the host myocardium. The cells were grown in multielectrode array (MEA) assemblies, which allowed application of stimulatory current pulses and recording of field action potentials (FAPs) propagating in the cell population using 60 electrodes (Figure 4AC). Spontaneous FAPs were observed in 100% (5 out of 5) of cultures of pure cardiac myocytes, 40% (4 out of 10) of co-cultures of connexin 43 transduced skeletal myoblasts with cardiac myocytes and 12.5% (1 out of 8) of co-cultures of non-transduced skeletal myoblasts with cardiac myocytes. In the latter case FAP activation had a sporadic pattern. Stimulation with 10 pulses of current applied during 10 s was sufficient to obtain FAPs in 100% (10 out of 10) of co-cultures of connexin 43 transduced skeletal myoblasts with cardiac myocytes and 25% (2 out of 8) of co-cultures of non-transduced skeletal myoblasts with cardiac myocytes. Again, in the latter case the pattern of FAP activation was always only sporadic. No FAP activation was observed in 10 individual cultures of non-tranduced skeletal myoblasts and 10 individual cultures of transduced skeletal myoblasts. Typical electrograms are presented in Figure 4BD (illustration for all 60 electrodes is shown in Additional file 1: Electrograms.pdf). The mean FAP activation rate for the 60 electrodes over 10 s after the last stimulation pulse was 2.74 ± 0.20 Hz for cardiac myocytes alone, 1.97 ± 0.34 Hz for co-culture of connexin 43 transduced skeletal myoblasts with cardiac myocytes (no significant difference compared with cardiac myocytes alone) and 0.44 ± 0.19 Hz for co-culture of non-transduced skeletal myoblasts with cardiac myocytes (P < 0.001 compared with cardiac myocytes alone, and P < 0.002 compared with co-culture of connexin 43 transduced skeletal myoblasts with cardiac myocytes).

Thus, a comparison of all the experimental groups indicated that electrical coupling in the co-culture of skeletal myoblasts with cardiac myocytes is significantly enhanced in the presence of overexpressed connexin 43.

## Discussion

Electric communication between cells is mediated by bursts of the action potential and gap junctions provide the low resistance pathway for its cell-to-cell propagation. In addition gap junctions mediate flux of small molecules that regulate normal tissue development and tissue patterning. It is, therefore, reasonable to hypothesise that gap junctions can play a central role in the electromechanical incorporation of cardiac grafts. Autologous skeletal myoblasts are an attractive source for cardiac transplants because of their immune privileges, availability and non-tumourogenicity (reviewed in [21]). However, when skeletal myoblasts differentiate into myotubes, they permanently lose expression of the major gap junctional protein, connexin 43, and, thus, do not have the apparatus for gap-junctional coupling. This is most likely to be the reason why, after transplantation of skeletal myoblasts, the newly developed graft has not been observed beating synchronously with the heart tissue [22]. There was no intercellular transfer and electrical coupling between the cells developed from the transplanted myoblasts and the host cardiac myocytes [23]. It was also reported that human patients with engrafted skeletal myoblasts suffered from ventricular tachycardia [24,25]. Therefore, in pursuit of improved cardiac integration of skeletal muscle grafts we modified primary skeletal myoblasts by overexpression of the main gap-junctional protein connexin 43.

We have chosen a retroviral MLV vector for delivery and overexpression of the connexin 43 transgene into skeletal myoblasts because retroviral vectors are able to integrate stably into the genome of the transduced cells and thus to provide long-term expression of a transgene [26]. Indeed, in our study, expression of connexin 43 and the EGFP marker in the transduced cells did not subside after 12 weeks of continuous passaging in tissue culture. However, long term gene expression *in vitro *can be only tentatively projected to long term gene expression *in vivo*, where transgene expression shutdown events are common [27]. Therefore, one should consider the possible need of 'topping up' connexin 43 gene expression in the transplanted tissue *in vivo*. As the engrafted cells do not actively divide, the choice of the 'topping up' vector is limited to cell division independent vectors, for example lentiviral HIV-based vectors. In this context employment of an MLV vector at the *ex vivo *transduction step is beneficial because it allows subsequent use of efficient and MLV-compatible lentiviral vectors for additional transduction of the grafts *in vivo*. If lentiviral vectors were used for initial transduction *ex vivo*, there would be a possibility of a reduced efficiency of the 'topping up' transgene delivery *in vivo *because of a CD4-independent superinfection interference of the resident lentiviral vector with the incoming one ([28], reviewed in [29]).

Absence of connexin 43 in adult muscle can be due to shutdown of the native connexin 43 promoter during myoblast differentiation. We chose the MLV LTR and the CMV promoters to drive expression of human connexin 43 cDNA because these viral promoters are known to be able to direct transcription in adult muscle and therefore they are unlikely to shut down due to changes in the balance of transcription factors in the course of myoblast differentiation. A tandem arrangement of the two promoters in the MLV-CX43-EGFP vector could reduce the chances of transcription silencing after transplantation.

The EGFP marker of the generated viral vector MLV-CX43-EGFP was useful for the purification of the transduced cells by FACS and it can also be useful at the post-grafting stage to track the transplants *in vivo*.

Our observations show that confluent cultures of primary myoblasts can stay alive for at least a month in medium supplemented with FCS (and considerably longer in medium without serum). This property of primary myoblasts was in stark contrast to the transformed rat L6 myoblasts, which often died in a week after achieving confluency. Thus, fortuitously, the number of culture passages (and, therefore, cell divisions) required for our manipulation of primarily myoblasts (magnetic sorting, retroviral transduction, preparative FACS) was lower, than the number of passages needed for analogous manipulations with a permanent myoblast cell line L6.

To increase the yield of connexin 43 transduced skeletal myoblasts from a single muscle biopsy it is important to achieve a high efficiency of transduction by the MLV vector. However, preparations of amphotropic MLV commonly contain infection inhibiting substances, which reduce maximal transduction efficiencies without reduction of end-point virus titres [17]. It is, therefore, important to optimise conditions for high efficiency of transduction. In our experiments we have shown for the first time that a combination of viral vector concentration on the plastic surface using the virus-binding protein RetroNectin^® ^[19] and transduction in the presence of lipid polycation Transfectam^® ^[20] is particularly effective for transduction of primary myoblasts by an amphotropic MLV vector. The achieved efficiency of transduction (38.30 ± 0.89%) can be further increased by: 1) improving the viral vector titre, for example by virion production at 32°C [30]; 2) additional concentration of the viral vector, e.g. by using magnetic nanoparticles [31] or low speed centrifugation [19]; 3) increasing transduction competence of the recipient cells, for example by phosphate starvation of the myoblasts [17] or boosting the myoblast division rate using growth factors [32].

We have demonstrated that connexin 43 transduction of skeletal myoblasts and ensuing connexin 43 overexpression significantly improves propagation of action potential (measured as FAP activation rate) in co-culture of cardiac myocytes and skeletal myoblasts *in vitro*. Enhanced gap junction formation between connexin 43 transduced skeletal myoblasts and cardiac myocytes is the most likely mechanism involved. This conjecture is supported by the results of Reinecke *et al *[33] who reported that transplantation of genetically engineered myoblasts, which were designed to express connexin 43 during differentiation, resulted in close apposition of the skeletal myotubes and the host cardiac myocytes.

Skeletal myoblasts are non-differentiated muscle cells and, unsurprisingly, we did not observe any FAP activation in pure cultures of skeletal myoblasts, whether overexpressing connexin 43 or not. Thus, although fluorescent dye transfer occurred to a significantly greater extent in connexin 43 transduced skeletal myoblasts, improved gap-junctional communication in these cells did not result in FAP generation. We registered only a limited FAP activation in co-cultures of non-transduced skeletal myoblasts with cardiac myocytes. However, with connexin 43 overexpression in the skeletal myoblasts, the FAP activation rate in the co-cultures of skeletal myoblasts and cardiac myocytes was significantly enhanced, and was close to the FAP activation rate in pure cultures of cardiac myocytes.

## Conclusion

More than 4 times overexpression of connexin 43 in primary skeletal myoblasts was achieved after retroviral transduction in optimised conditions involving a concomitant use of the retrovirus immobilising protein RetroNectin^® ^and the polycation transduction enhancer Transfectam^®^. Connexin 43 overexpression resulted in improvement of electrical coupling between transduced skeletal myoblasts and cardiac myocytes *in vitro*. Thus, retroviral connexin 43 transduction is a useful step for engineering of electrocompetent cardiac grafts.

## Abbreviations

Ap – ampicillin, ASE – average standard error, Cm – chloramphenicol, DMEM – Dulbecco's modified Eagle's medium, DMSO – dimethylsulphoxide, EDTA – ethylenediaminetetraacetic acid, EGFP – enhanced green fluorescence protein, FACS – fluorescence activated cell sorting, FAP – field action potential, FCS – fetal calf serum, CMV – cytomegalovirus, HIV – human immunodeficiency virus, IRES – internal ribosome entry site, KF – Klenow fragment of *Escherichia coli *DNA polymerase I, LTR – long terminal repeat, M – mean, MEA – multielectrode array, MLV – murine leukemia virus, PBS – phosphate buffered saline, PCR – polymerase chain reaction, rpm – revolutions per minute, SDS-PAGE – sodium dodecyl sulfate polyacrylamide gel electrophoresis, SE – standard error, TU – transduction unit.

## Competing interests

The authors declare that they have no competing interests.

## Authors' contributions

OT generated the connexin 43 retroviral vector, supervised and performed primary myoblast isolation, retroviral transductions, cell sorting and longevity study of EGFP expression, took part in Western blotting analysis of connexin 43 expression, drafted the manuscript. YM isolated primary myoblasts, transduced them with a retroviral vector, performed dye injection and multielectrode array experiments, drafted the manuscript. PP performed Western blotting analysis. HS participated in MEA experiments. KTM and NDU performed some dye injections experiments. YK performed some of the experiments in longevity study of EGFP expression. MT, CC and NSP conceived the study and revised the manuscript. All authors read and approved the final manuscript.

## Pre-publication history

The pre-publication history for this paper can be accessed here:



## Supplementary Material

Additional File 1 **Multi-electrode array recording in co-cultures of primary skeletal myoblasts with cardiac myocytes (data for all 60 electrodes)**. Recordings from 60 electrodes in the MEA are presented as a collection of 60 individual electrograms (x-axis – time in s, y-axis – potential in μV). The time window frame was chosen to show the last stimulatory current pulse (in the series of 10, delivered with the frequency of 1 Hz). **(A) **A nest of electrograms showing the last stimulatory pulses and absence of any ensuing FAP spikes in co-cultures of cardiac myocytes with non-transduced skeletal myoblasts (recordings from all 60 electrodes of the MEA). **(B) **A nest of electrograms showing the last stimulatory pulses and the ensuing FAP spikes in co-cultures of cardiac myocytes with connexin 43 transduced skeletal myoblasts. Click here for file

Additional File 2 **Immunoconfocal analysis of a myogenic marker desmin in populations of primary myoblasts at an early stage and a late stage of cultivation**. Immunostaining was performed with anti-desmin mouse monoclonal antibody as a primary antibody and goat anti-mouse Cy3-labelled as a secondary antibody. Cells were grown to form a monolayer on glass cover slips and were fixed with ice-cold methanol before immunostaining. Images were obtained using a Leica TCSNT confocal microscope at an instrumental magnification of 800 times. Phase contrast **(A) **and immunostaining **(B) **micrographs of primary rat myoblasts obtained after magnetic sorting with anti-α-7 integrin antibody. Phase contrast **(C) **and immunostaining **(D) **micrographs of primary rat myoblasts after magnetic sorting with anti-α-7 integrin antibody followed by an additional 4-week passaging to allow transduction with the MLV-CX43-EGFP vector and EGFP-based preparative FACS sorting. Phase contrast **(E) **and immunostaining **(F) **micrographs of NIH3T3 mouse fibroblasts (desmin-negative control). Phase contrast **(G) **and immunostaining **(H) **micrographs of L6 rat myoblasts (desmin-positive control). Click here for file
