# Supplementary figures and images for: Overexpression of connexin 43 using a retroviral vector improves electrical coupling of skeletal myoblasts with cardiac myocytes in vitro
Source: BMC Cardiovasc Disord. 2006 Jun 6;6:25. doi: 10.1186/1471-2261-6-25 (PMC1513252; doi:10.1186/1471-2261-6-25)

A

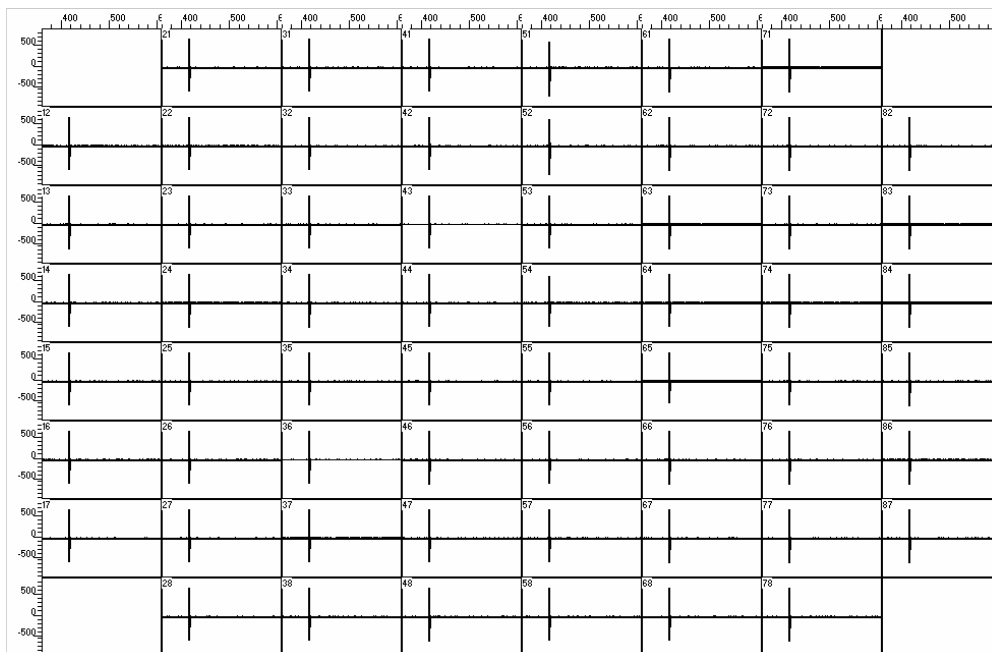

B

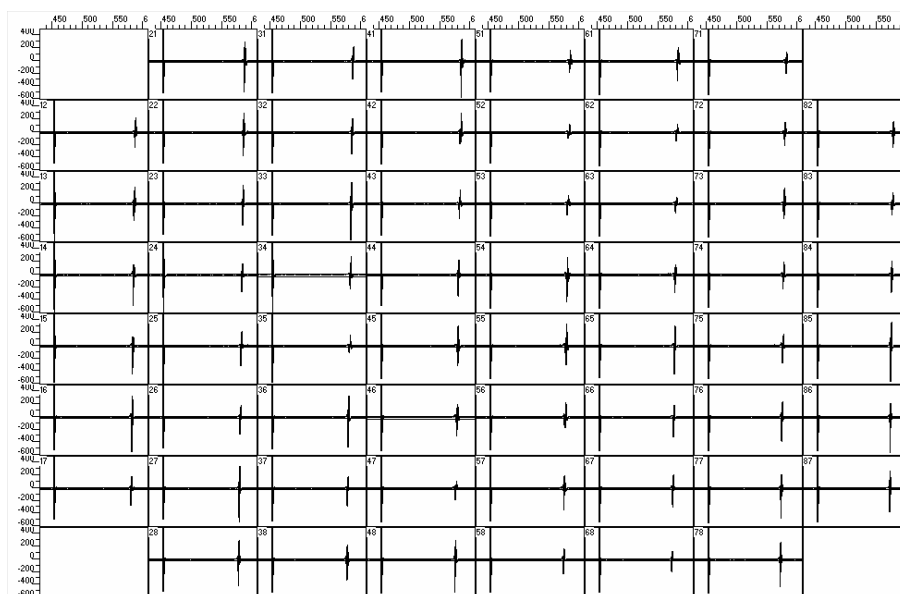

Supplement: Additional File 1 — Multi-electrode array recording in co-cultures of primary skeletal myoblasts with cardiac myocytes (data for all 60 electrodes). Recordings from 60 electrodes in the MEA are presented as a collection of 60 individual electrograms (x-axis – time in s, y-axis – potential in μV). The time window frame was chosen to show the last stimulatory current pulse (in the series of 10, delivered with the frequency of 1 Hz). (A) A nest of electrograms showing the last stimulatory pulses and absence of any ensuing FAP spikes in co-cultures of cardiac myocytes with non-transduced skeletal myoblasts (recordings from all 60 electrodes of the MEA). (B) A nest of electrograms showing the last stimulatory pulses and the ensuing FAP spikes in co-cultures of cardiac myocytes with connexin 43 transduced skeletal myoblasts. [file 1471-2261-6-25-S1.pdf]

A

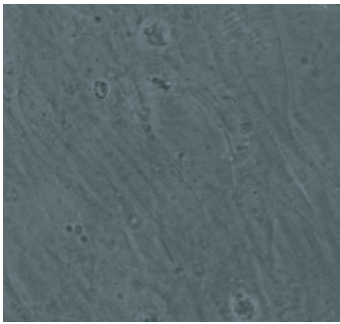

B

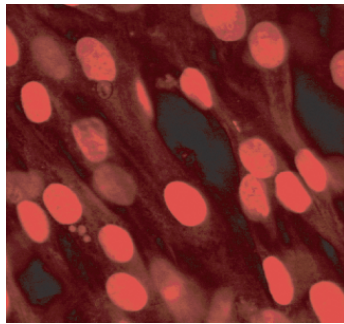

C

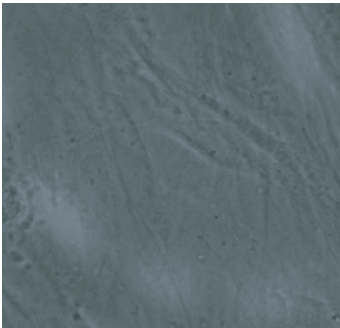

D

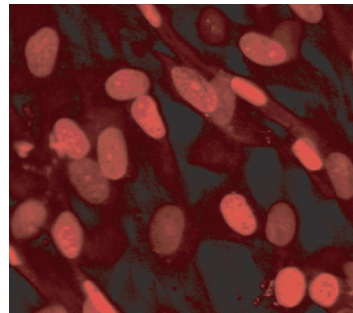

E

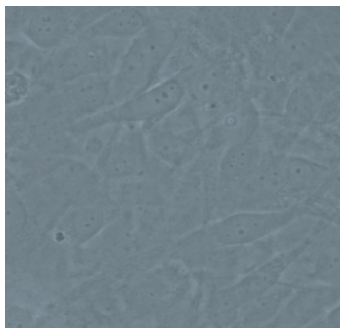

F

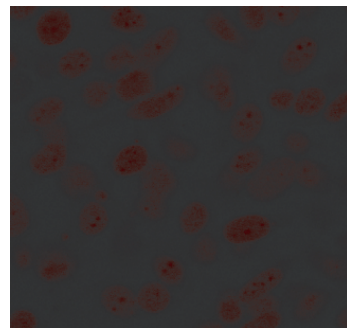

G

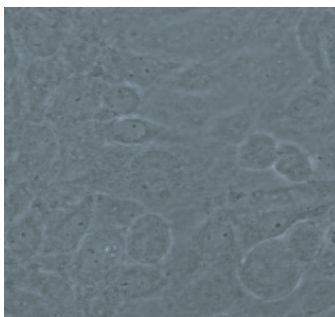

H

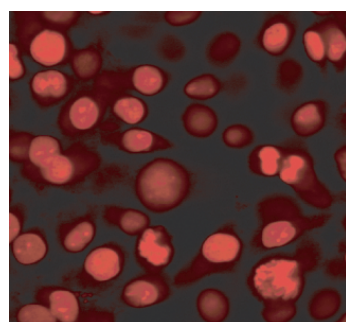

Supplement: Additional File 2 — Immunoconfocal analysis of a myogenic marker desmin in populations of primary myoblasts at an early stage and a late stage of cultivation. Immunostaining was performed with anti-desmin mouse monoclonal antibody as a primary antibody and goat anti-mouse Cy3-labelled as a secondary antibody. Cells were grown to form a monolayer on glass cover slips and were fixed with ice-cold methanol before immunostaining. Images were obtained using a Leica TCSNT confocal microscope at an instrumental magnification of 800 times. Phase contrast (A) and immunostaining (B) micrographs of primary rat myoblasts obtained after magnetic sorting with anti-α-7 integrin antibody. Phase contrast (C) and immunostaining (D) micrographs of primary rat myoblasts after magnetic sorting with anti-α-7 integrin antibody followed by an additional 4-week passaging to allow transduction with the MLV-CX43-EGFP vector and EGFP-based preparative FACS sorting. Phase contrast (E) and immunostaining (F) micrographs of NIH3T3 mouse fibroblasts (desmin-negative control). Phase contrast (G) and immunostaining (H) micrographs of L6 rat myoblasts (desmin-positive control). [file 1471-2261-6-25-S2.pdf]
